# Supplementary material for: Monolithic lithium niobate photonic circuits for Kerr frequency comb generation and modulation
Source: Nat Commun. 2019 Feb 28;10:978. doi: 10.1038/s41467-019-08969-6 (PMC6395685; doi:10.1038/s41467-019-08969-6)
Supplement: Supplementary file 1 — Supplementary Information [file 41467_2019_8969_MOESM1_ESM.pdf]

Supplementary Information for

Monolithic lithium niobate photonic circuits for Kerr frequency comb generation and modulation

Wang et al.

This PDF file includes:

Supplementary Figure 1 to 5 and Legends

Supplementary Note 1 to 6

Supplementary Note 1 | Optical parametric oscillation threshold

The threshold power of the OPO process can be estimated using Supplementary Equation 1 [1]:

$$P_{\mathrm{th}} \approx 1.54\left(\frac{\pi}{2}\right)\frac{Q_{\mathrm{C}}}{2Q_{\mathrm{L}}}\cdot\frac{{n_{\mathrm{eff}}}^{2}V}{n_2\lambda_{\mathrm{p}}{Q_{\mathrm{L}}}^{2}} \tag{1}$$

where Q_C and Q_L are the coupling and loaded Q factors of the resonator, n_{eff} is the effective refractive index of the lithium-niobate (LN) waveguide, n_2 is the nonlinear refractive index, λ_p is the pump wavelength and V is the resonator mode volume.

From the measured loaded quality (Q) factor $Q_L = 6.6 \times 10^5$ and the optical transmission depth T = 4.6% for transverse-electric (TE) mode (Fig. 1b), we estimate the intrinsic quality factor $Q_{\mathrm{i}} = \frac{2Q_{\mathrm{L}}}{1+\sqrt{T}} = 1.1 \times 10^6$ assuming the device is under-coupled. Since $Q_L^{-1} = Q_C^{-1} + Q_i^{-1}$, the coupling Q is calculated to be $Q_C = 1.7 \times 10^6$. We numerically calculate the effective index of our LN waveguide to be $n_{\mathrm{eff}} = 1.91$ and the mode area $A = 0.875$ μm^2. The resonator mode volume is $V = 2\pi RA = 440$ μm^3, where $R = 80$ μm is the radius of the ring resonator. The nonlinear refractive index $n_2 = 0.91 \times 10^{-15}$ cm^2 W^{-1}. From these parameters we calculate the optical parametric oscillation threshold to be $P_{\mathrm{th}} = 80$ mW.

Supplementary Note 2 | Relative intensity noise

To measure the relative intensity noise (RIN) of the generated Kerr combs, we filter out the comb lines from 1580 nm to 1700 nm using a homebuilt *4f* shaper and send to a photodetector (Newport 1811, 3-dB bandwidth: 125MHz). RIN spectra are obtained by analyzing the photodetector output signals using a real-time signal analyzer (Tektronix RSA 5126A, resolution bandwidth: 100 kHz, video bandwidth: 1 kHz). Supplementary Figure 1 shows the measured RIN spectra, which

indicates both combs are in the high-noise (modulation instability) state.

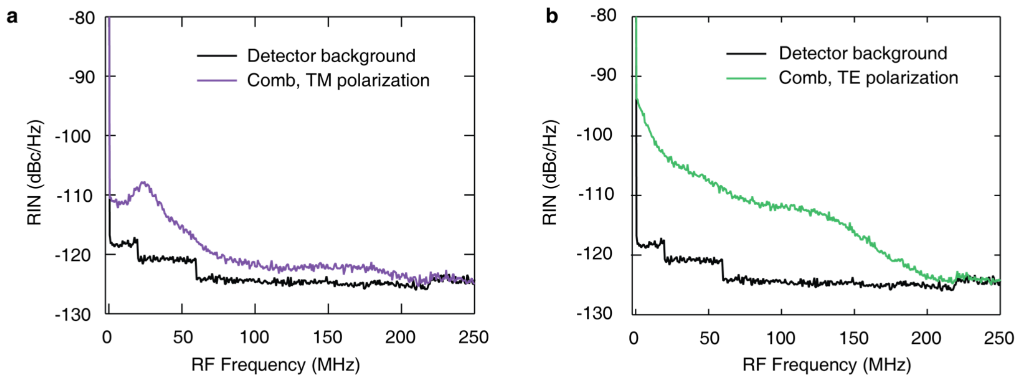

Supplementary Figure 1 | Relative intensity noise spectra of the combs pumped at TM polarisation (a) and TE polarisation (b). Comb lines from 1580 to 1700 nm are filtered out for the measurement.

Supplementary Note 3 | Raman effects in the comb generation process

We identify that the spur-like features in the generated comb spectra could be attributed to corresponding Raman modes of LN crystal. Supplementary Figure 2 shows the locations of the spur-like features in the comb spectra and their corresponding Raman modes for both TM- and TE-polarised frequency combs [2]. Each corresponding Raman mode is labelled with its symmetry and frequency shift. Both E and A mode are Raman-active optical phonon branches in LN. TO and LO correspond to transverse and longitudinal optical modes, respectively. The peak around 1605 nm in Supplementary Figure 2b is attributed to modulation instability. In future work, Raman effects could potentially be suppressed by engineering the resonator FSR to avoid the Raman modes [3], therefore to facilitate the generation of soliton states.

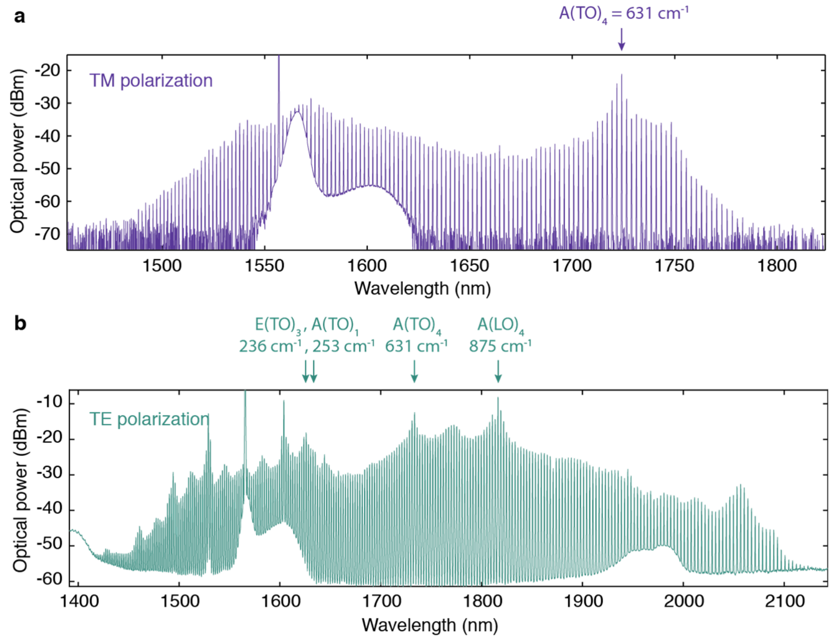

Supplementary Figure 2 | Frequency comb spectra with corresponding Raman modes. The spur-like features in both TM-polarised (a) and TE-polarised (b) comb spectra could be attributed to corresponding Raman modes, as are labeled in the figure.

Supplementary Note 4 | Photorefractive instability

Within LN microresonators, the photorefractive effect blue shifts the resonance while thermal effect induces a red shift. At certain power levels, competition between the two effects could cause instability of the resonance frequency, the mechanism of which was studied in detail by X. Sun et al [4]. Supplementary Figure 3 shows the measured photorefractive instability at various input power levels for our resonators. We measure such effects by sweeping the laser frequency in both directions. At low optical powers (< 100 µW), since both nonlinear effects are weak, both forward and backward sweeps show Lorentzian-shaped resonance dips with the same linewidth, therefore ensuring the measured Q factors are accurate (Fig. 1b). At medium optical powers (100 µW – 50 mW), the competition between photorefractive and thermal effects results in a sharp resonance dip

when sweeping from blue to red, and a much broadened, oscillatory response when sweeping from red to blue (Supplementary Figure 3a-b). Such behaviour could cause difficulties in stabilizing the cavity within resonance. When the optical power further increases to > 50 mW, the oscillation becomes longer in period and eventually is suppressed due to the dominance of thermal bistability (Supplementary Figure 3c). The quenching of photorefractive instability at high powers allow us to stably position the laser detuning with respect to cavity resonance and achieve stable Kerr comb generation. The measured on-resonance optical transmission levels are higher than that in Fig. 1b due to the presence of amplified spontaneous emission (ASE) noise from the erbium-doped fibre amplifier (EDFA).

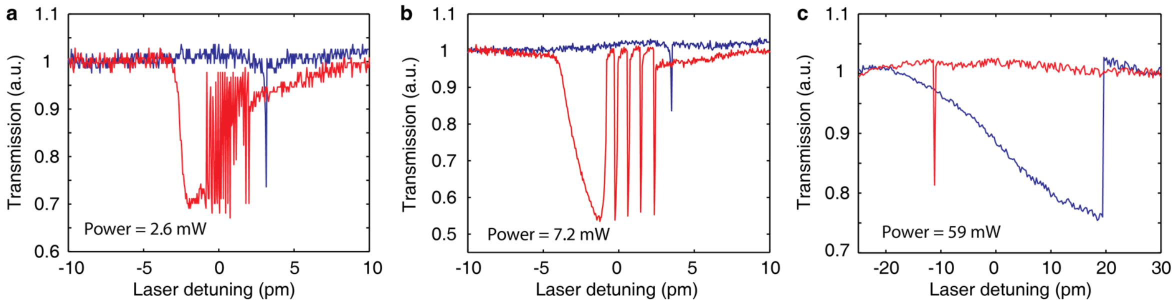

Supplementary Figure 3 | Photorefractive instability. Transmission spectra of the cavity resonance when increasing the optical powers in the bus waveguide from 2.6 mW (a) to 7.2 mW (b) and eventually to 59 mW (c). The transmission spectra are measured by sweeping the laser frequency in both forward (blue curves) and backward (red curves) directions. The laser sweeping speeds are kept at 1 nm s^{-1}.

Supplementary Note 5 | Filter transmission spectra

The filter ring resonator is designed to have a free spectral range (FSR) that is 1.2% larger than the comb-generating resonator. Supplementary Figure 4 shows the optical transmission spectra at both

through and drop ports of the filter, measured using a tunable telecom laser. The transmission dips at the through port correspond to resonances of both ring resonators, while the transmission peaks at the drop port correspond to the filter passbands. The spectra show that the two ring resonances are aligned near ~ 1606 nm, and have increasing mismatch when the wavelength is away from 1606 nm.

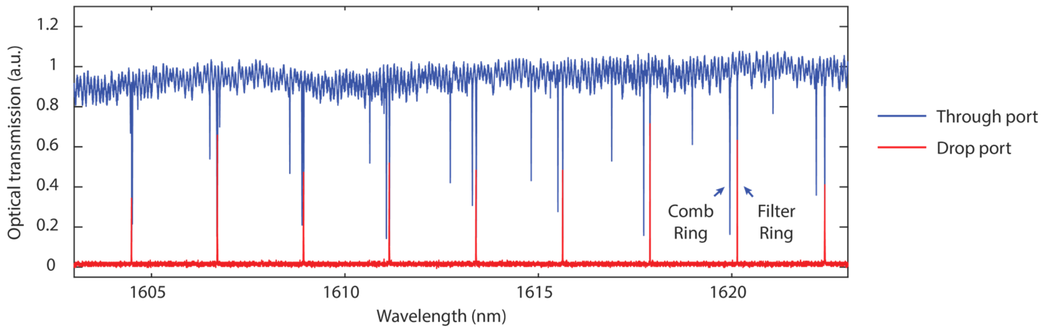

Supplementary Figure 4 | Optical transmission spectra of the device at both through and drop ports of the filter. The resonances of the comb generator and the filter are aligned near 1606 nm, and have a 1.2% mismatch in the free spectral range.

Supplementary Note 6 | Filter transfer function

Supplementary Figure 5 shows the theoretical transfer function of the filter, which has a FSR of 2.19 nm and a linewidth of 3 pm. The calculated suppression ratio for the next comb line, which has a 27-pm resonance mismatch, is 25 dB. The calculated suppression ratio for the pump light (resonance mismatch by 730 pm) is 52 dB.

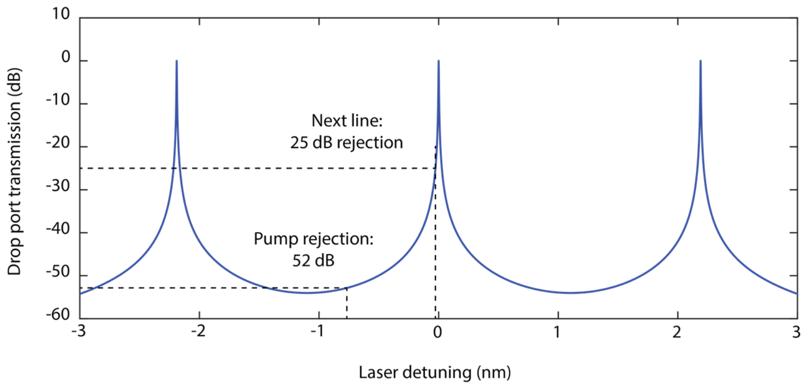

Supplementary Figure 5 | Numerically calculated filter transfer function. The calculated suppression ratios for the next line and the pump are 25 dB and 52 dB respectively.

Supplementary References

1 Kippenberg, T. J., Spillane, S. M. & Vahala, K. J. Kerr-nonlinearity optical parametric oscillation in an ultrahigh-Q toroid microcavity. *Phys. Rev. Lett.* **93**, 083904 (2004).

2 Kaminow, I. P. & Johnston, W. D. Quantitative Determination of Sources of the Electro-Optic Effect in LiNbO3 and LiTaO3. *Phys. Rev.* **160**, 519-522 (1967).

3 Okawachi, Y. *et al.* Competition between Raman and Kerr effects in microresonator comb generation. *Opt. Lett.* **42**, 2786-2789 (2017).

4 Sun, X. *et al.* Nonlinear optical oscillation dynamics in high-Q lithium niobate microresonators. *Opt. Express* **25**, 13504-13516 (2017).
